# Effects of Slaughter Age of Holstein Friesian Bulls on Meat Quality: Chemical Composition, Textural Characteristics, Sensory Attributes and Fatty Acid Profile

**DOI:** 10.3390/foods12010158

**Published:** 2022-12-28

**Authors:** Abdulkerim Diler, Mete Yanar, Veysel Fatih Özdemir, Recep Aydin, Özgür Kaynar, Valiollah Palangi, Maximilian Lackner, Rıdvan Koçyigit

**Affiliations:** 1 Department of Plant and Animal Sciences, Vocational School of Technical Sciences, Ataturk University, 25240 Erzurum, Turkey; 2 Department of Animal Science, College of Agriculture, Ataturk University, 25240 Erzurum, Turkey; 3 Department of Biochemistry, Faculty of Veterinary Medicine, Kastamonu University, 37150 Kastamonu, Turkey; 4 Department of Animal Science, Agricultural Faculty, Ataturk University, 25240 Erzurum, Turkey; 5 Department of Industrial Engineering, University of Applied Sciences Technikum Wien, Hoechstaedtplatz 6, 1200 Vienna, Austria

**Keywords:** slaughter age, texture profile analysis, sensory evaluation, fatty acid profile

## Abstract

This study aimed to investigate the effects of slaughter age (young vs. old), muscle type (Longissimus dorsi (LD), Gluteus medius (GM)) and fat deposits (kidney knob and channel fat, subcutaneous fat, intramuscular fat) on chemical, organoleptic, textural characteristics and fatty acid composition of Holstein Friesian bull meat. For this purpose, the carcasses of 26 Holstein Friesian bulls that had been fattened on the same private farm were assigned to two experimental groups based on their age at slaughter: a young group (YG) (average age: 17.0 ± 1.0 months old) and an old group (OG) (average age: 22.0 ± 1.0 months old). The percentage of crude protein, panel tenderness score, polyunsaturated fatty acid (PUFA) and saturated fatty acid (SFA) content, the PUFA/SFA ratio and the hypocholesterolemic fatty acid (DFA)/hypercholesterolemic fatty acid (OFA) ratio of the bull carcasses decreased significantly with increasing slaughter age. By contrast, the OFA content of the carcasses significantly increased (*p* < 0.05) with increasing slaughter age. Advanced slaughter age resulted in lower panel tenderness scores. Additionally, the meat of the bulls in the OG was considered to be less healthy because of the less desirable fatty acid composition and nutritional indices, such as the PUFA/SFA and hypocholesterolemic/hypercholesterolemic ratios, compared to the meat from the bulls in the YG. Furthermore, the intramuscular fat and internal fat contained high percentages of PUFA and SFA and high PUFA/SFA and hypocholesterolemic/hypercholesterolemic ratios. Interestingly, the percentage of OFA content in the internal and intramuscular fat tissues decreased with increasing slaughter age. In conclusion, this study provided evidence that slaughter age and muscle and fat type are essential sources of variations in the textural characteristics, sensory panel attributes and fatty acid profile of meat from Holstein Friesian bulls.

## 1. Introduction

Red meat, i.e., meat from certain livestock animals (such as beef), is a vital part of the human diet and it contains desired nutritional qualities, including the high biological values of proteins and the high content of vitamins (A, B6, B12, D and E), minerals (Fe, Se and Zn) and essential fatty acids (especially α-linoleic, eicosapentaenoic and docosapentaenoic acids) [1]. Ozdemir and Yanar [2] investigated red meat consumption among Turkish people and found that beef consumption ranked first in the country and that the share of cattle within the total red meat production was 89.5%. In recent years, increasing income and educational levels among meat consumers in many countries, including Turkey, have led to increased demand for high-quality and healthy meat. Additionally, meat consumers have also become increasingly health-conscious [3]. On the other hand, the variations in beef quality are very high and inconsistent, and the origins of that variability are often multi-factorial [4]. In particular, consumers judge the quality of meat based on its dietetic, appearance and organoleptic properties [5]; if they are unsatisfied with these properties, meat consumers may reject purchasing them [6]. Due to this, better control of meat quality to satisfy consumer demand for meat and meat products has become a significant issue for beef producers and retailers over recent decades. Therefore, many studies have focused on investigating the chemical and fatty acid composition of meat in an attempt to improve its quality and sensory attributes [7].

The composition of lipids in meat includes a considerable amount of SFA, which is responsible for increasing LDL (low-density lipoprotein) cholesterol; in turn, LDL cholesterol has the adverse effect of the formation of coronary heart diseases. On the other hand, the predominant fatty acid in beef is oleic acid (C18:1 (n−9)), which is a monounsaturated fatty acid (MUFA). Unsaturated fatty acids (UFAs) constitute approximately 50% of the total fatty acids [8]. Health organizations recommend reducing the amount of SFA and total fat and increasing the PUFA/SFA ratio in human diets to protect cardiovascular health [9]. The fatty acid composition of ruminant tissues is less determined by their ration as a large amount of the PUFA in their diet is converted into more saturated forms by microbial metabolism in the rumen. However, slaughter age could affect the subcutaneous fat, internal fat and intramuscular fat profiles of carcasses with subsequent alterations in the levels of SFA, MUFA and PUFA [10].

Holstein Friesian cattle that are reared on the elevated plains of Eastern Turkey have characteristic morphological anatomies and are relatively smaller, in terms of both body weight and size, than their counterparts that are raised in lowland European countries [2]. Although Holstein Friesian cattle reared in this region have become smaller, they are quite well adapted to the harsh environmental conditions in the eastern part of the country, where a wide range of climate patterns are seen [11]. There is scarce information about the implications of the different slaughter ages of bulls on the properties of their meat, i.e., its textural characteristics, fatty acid composition and sensory attributes, particularly with regard to Holstein Friesian cattle that are reared in harsh climatic and geographical conditions in Eastern Turkey. Therefore, this study was undertaken not only to investigate the effects of age (upon slaughter) and muscle type on chemical, organoleptic and textural characteristics of meat from Holstein Friesian bulls but also to determine the effects of slaughter age and various fat deposits on the fatty acid composition of the meat. The bulls in this study were allocated into two groups: young group (YG) comprising 17-month-old bulls and an old group (OG) comprising 22-month-old bulls. The typical slaughter age of bulls is reported to be between 12 and 22 months, whereas their expected natural life span is 15–20 years.

## 2. Materials and Methods

### 2.1. Experimental Animals and Experimental Design

This work was conducted experimentally in the Department of Animal Science at the College of Agriculture, Erzurum, Turkey (39°54′05″ N; 41°14′15″ E). The research subjects were the carcasses of 26 Holstein Friesian bulls that had been fattened on the same private farm in Erzurum Province, Eastern Turkey. The animals were fed a total mixed ration (TMR) via group feeding during the fattening period. The animals were fed ad libitum TMR that consisted of 30% dry hay and 70% dry matter concentrate. The chemical composition of the concentrate used in this study was 85.2% dry matter, 18.0% crude protein, 7.7% crude ash, 2.3% ether extract, 40.5% neutral detergent fiber and 21.4% acid detergent fiber. The dry hay contained 92.8% dry matter, 9.1% crude protein, 8.8% crude ash, 2.8% ether extract, 63.7% neutral detergent fiber and 39.1% acid detergent fiber.

Prior to slaughter, the bulls in this study were assigned into two groups based on their slaughter age: the young group (YG) (average: 17.0 ± 1.0 months old; n = 13) and the old group (OG) (average: 22.0 ± 1.0 months old; n = 13). After the slaughtering process, the carcasses of the bulls were transported from the Erzurum Meat and Milk Board abattoir. The slaughter and post-slaughter procedures in the abattoir were carried out in line with the slaughter and carcass preparation procedures of the Turkish Standards Institute [12].

From the fresh carcasses, which were cleaved along the spine, two parts were obtained and cooled in a commercial chiller at 4 °C for 24 h. After 24 h, the carcasses were ribbed at the 12–13 rib interface.

### 2.2. Preparation of Samples

Twenty-four hours after slaughter, LD and GM muscle samples from 26 carcasses (YG:13, OG:13) were obtained. The sample size of the study was determined by using G*Power 3.1.9.7 statistical software. Four kilograms of meat samples from the *Gluteus medius* (GM), which is a gluteal muscle on the outside of the pelvis, and four kilograms of meat samples from the *Longissimus dorsi* (LD), which is a large flat dorsolateral muscle, were excised from the right-hand lumbar section of the carcasses. The GM muscle samples were cut into three slices that were perpendicular to the fiber direction, which was then used for our textural profile analysis (TPA) and chemical composition and sensory evaluations. However, the LD muscle samples were sectioned into 4 equal slices for TPA and chemical composition, intramuscular fatty acid composition and sensory evaluation. Six, five and eight subsamples of GM and LD muscles, respectively, were used for textural profile analysis with WBSF measurements, chemical composition and sensory evaluation. Additionally, 3 subsamples of LD muscle were allocated for the determination of the fatty acid composition of intermuscular fat. In order to determine the effects of other fat deposits on the fatty acid composition, about 100 g of internal fat samples (kidney knob and channel fat) and 100 g of subcutaneous fat samples were taken. The subcutaneous fat was taken from back fat over the LD, which was located on the first and second lumbar vertebrae. The muscle and fat samples were individually labeled. The animal number, muscle name, fat type and slaughter age group were annotated on the identification tag. The samples were then placed in polyethylene zipper bags and kept in a deep freezer at −20 °C for 15 days.

### 2.3. Sensory Evaluation

Before cooking, the frozen samples were taken out of the deep freezer and immediately placed in a refrigerator. The frozen meat samples were thawed at 4 °C in the refrigerator for 18 h, as described by Akkose [13]. A water bath was used for cooking the samples homogeneously. The meat specimens were put into water impermeable polyethylene bags, completely immersed in the water bath at a constant temperature of 90 °C and cooked until their internal temperature reached 70 °C. The internal temperature of the meat samples was monitored using a meat thermometer. After cooking, the samples were removed from the water bath and cooled under running tap water. The meat samples were then removed from the bags. In order to remove excess fluid, the cooked meat samples were put onto a paper towel for approximately 5 min. The cooked samples from both the LD and GM were subsequently cut into uniform pieces (approximately 1 × 1 × 1 cm³). The cooked samples were randomly distributed to eight panelists, who had been instructed to evaluate the samples using the following properties: tenderness, juiciness, flavor intensity, palatability and general acceptability. For this purpose, they used nine-point hedonic scales (1 = extremely tough to 9 = extremely tender; 1 = extremely dry to 9 = extremely juicy; 1 = extremely weak beef flavor to 9 = extremely strong beef flavor; 1 = extremely poor to 9 = extremely palatable; 1 = extremely low acceptability to 9 = extremely high general acceptability). The number of chews required before swallowing was also determined by counting and then recorded.

### 2.4. Textural Properties

#### 2.4.1. Warner–Bratzler Shear Force Measurement

Cooked meat samples were used to determine the Warner–Bratzler Shear Force (WBSF) values. The samples were cooled to 20 °C, and then six 1.3 cm pieces were removed, which were parallel to the longitudinal orientation of the muscle fibers. They were cut twice using a WBSF device (G.R. Electricals Manufacturing Co., Topeka, KS, USA), which was equipped with a stainless steel blade (1.18 mm in thickness and 126.77 mm in height) that had a V-shaped (60° angle) cutting edge and a capacity of 25 kg at a velocity of 20 cm/min. The WBSF values were then measured as lb [14].

#### 2.4.2. Textural Profile Analysis

A texture analyzer (CT3, Brookfield Engineering, USA) was used for the TPA. For this purpose, the meat samples were cooked (as explained in Section 2.3.) and cooled to 20 °C. Then, cylindrical meat samples (20 mm in diameter and 20 mm in height) were analyzed over two compression cycles using a cylindrical probe with a 50.8 mm diameter (TA 25/1000, Brookfield Engineering). The settings used were as follows: pre-test speed = 1 mm/s; test speed = 2 mm/s; post-test speed = 2 mm/s; target strain = 50%; recovery time = 5 s. The textural profile analysis parameters, such as hardness (N), adhesiveness (mJ), cohesiveness, springiness (mm), chewiness (mJ), gumminess (N) and resilience, were determined force-time plots for the meat samples, as reported by Bourne [15].

### 2.5. Determination of Fatty Acid Profile

The samples that were taken from the LD muscle (intramuscular fat) and the internal fat (kidney knob and channel fat), and subcutaneous fat deposits from each carcass were analyzed 24 h post-mortem to determine the fatty acid profiles. For this, 1 g from each of the samples was mixed with 4 mL of SDS (sodium dodecyl sulfate, 10% in water) and then homogenized at 5000 rpm for 2 min using a tissue homogenizer. All samples were kept in an ice bath during the homogenization. The fatty acid composition of the samples was determined using high-performance thin-layer chromatography. First, the lipids were extracted from the samples by adding 500 μL of an n-hexane:isopropanol 3:2 (*v*/*v*) mixture to 1000 μL of meat homogenate [16] in an Eppendorf tube. The results obtained were the percentages of individual lipid classes out of the total lipid composition of the samples. The fatty acid (FA) profiles were evaluated as percentages of the total FA content (g/100 g of total fatty acids) [17].

### 2.6. Statistical Analysis

All statistical analyses were carried out using the SPSS statistic program (version 20). Prior to our statistical comparisons of the means of the main effects, all data were subjected to normality tests. The results of the Shapiro–Wilk normality test revealed that the data in this study had a normal distribution. Therefore, the data for our sensory panel evaluation, TPA and proximate analysis were statistically analyzed using a statistical model that included slaughter age and muscle type as the main effects. The interactions between slaughter age (YG and OG) and muscle type (LD and GM) were excluded from the statistical model since significant interactions were not identified in the preliminary statistical analysis. Another statistical model was then used to analyze the effects of slaughter age and fat type on fatty acid composition. The second statistical model included slaughter age (YG and OG) and fat type (subcutaneous fat, internal fat and intramuscular fat) as the main effects. Interactions between these effects were not included in our statistical analysis of the fatty acid profiles since those interactions were also not significant, according to the preliminary statistical analysis. Duncan’s multiple comparison test was used to compare the means when the results for the F test of the main effects were significant (with a significance level of α = 0.05).

## 3. Results

The least squares method was used to determine the means and standard errors of the chemical composition of the LD and GM muscle samples from bulls in the YG and OG, which are presented in Table 1. Although the differences in the chemical composition parameters were not significantly affected by the slaughter age (except for the percentage of crude protein), the different muscle types resulted in significant variations in the crude protein, crude ash (*p* < 0.01) and moisture (*p* < 0.05) content. The increase in protein level in the OG could be attributed to a decrease in the moisture content.

The sensory panel scores and the number of chews before swallowing for the samples are reported in Table 2. All of the organoleptic parameters, except for tenderness, were significantly influenced by slaughter age. However, muscle type significantly affected the juiciness and acceptability panel scores.

The data regarding the TPA parameters and WBSF measurements are provided in Table 3. Slaughter age did not have statistically significant effects on any parameters, including the WBSF value; however, the hardness, gumminess and chewiness TPA parameters were significantly (*p* < 0.01) influenced by muscle type.

Chewiness refers to the sensation in the mouth that is caused by the elastic resistance of food [19]. The WBSF (Warner–Bratzler Shear Force) measures meat tenderness [20].

**Figure 1:**
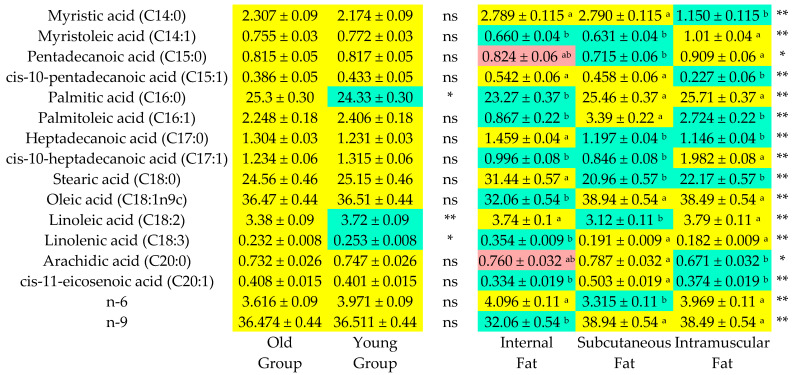
Heatmap plot of fatty acids composition of different fat types in carcasses of bulls depending on slaughter age. *: *p* < 0.05; **: *p* < 0.01; ns: non-significant; ^a,b^: Values in rows with different letters and colors differ sig.

**Figure 2:**
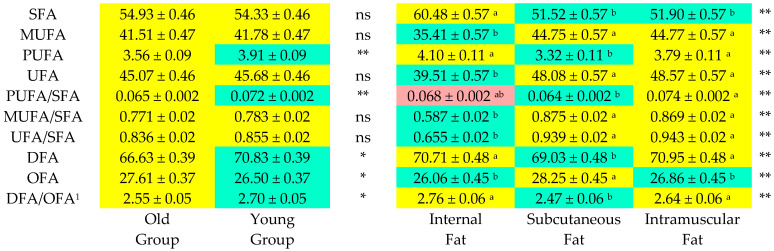
Heatmap plot of fatty acids profile of different fat types of the carcasses of bulls depending on slaughter ages. ^1^ DFA/OFA: hypocholesterolemic/hypercholesterolemic; *: *p* < 0.05; **: *p* < 0.01; ns: non-significant; ^a,b^: Values in rows with different letters and colors differ sig.

The least squares method was used to determine the means and standard errors for the fatty acid composition of internal fat, subcutaneous fat and intramuscular fat from the bulls, which are presented in Figure 1 and Figure 2. OFAs are fatty acids (C14:0 + C16:0) that have an undesirable hypercholesterolemic effect in humans, and DFAs (hypocholesterolemic fatty acids) are fatty acids (UFA + C18:0) that have a desirable hypocholesterolemic effect in humans. Both the OFA content and DFA content were significantly (*p* < 0.05) affected by slaughter age (*p* < 0.05) and fat type (*p* < 0.01). Furthermore, slaughter age (*p* < 0.05) and fat type (*p* < 0.01) also had a significant impact on the DFA/OFA (hypocholesterolemic/hypercholesterolemic) ratio. Although the PUFA and the PUFA/SFA ratio were significantly affected (*p* < 0.05) by slaughter age, the fat type was a significant (*p* < 0.01) source of variations in the SFA, MUFA, PUFA, UFA, UFA/SFA, MUFA/SFA and PUFA/SFA parameters.

## 4. Discussion

Consumers believe that quality meat should have the optimum nutritional composition. In this work, the implications of slaughter age on the percentages of crude ash and moisture in the muscle samples were not statistically significant, but the crude protein content was significantly affected by advanced slaughter age (Table 1). The percentage of protein in the meat samples from the bulls in the OG was 2.39% higher than that in samples from the bulls in the YG. Additionally, the crude fat content of the meat samples from the bulls in the OG was numerically greater than that of the bulls in the YG. In agreement with the results from this study, Bures and Barton [21], Nogalski et al. [22] and Pogorzelska-Przybyłek et al. [23] all reported that LD fat content increased and moisture content decreased as slaughter age increased. In the present study, muscle type had significant effects on all considered parameters, with the exception of the percentage of crude fat (Table 1). The measured parameters were higher in the LD samples than the GM samples, except for moisture content, which was higher in the GM samples. The results concerning the significant effects of muscle type were comparable to the findings reported by Russo and Preziuso [24], Ozluturk et al. [25] and Kopuzlu et al. [3].

Although there were a slight tendency for the juiciness, flavor intensity, acceptability and palatability scores to be higher in the YG, the effects of slaughter age on the sensory panel results were not significantly different, except for the tenderness score. One of the beef’s most significant organoleptic characteristics is tenderness, which has been found to exhibit the most pronounced effect on consumer satisfaction (provided that the panelists were representative of the consumer base). The tenderness score of the samples from the bulls in the YG was 9.0% higher than that of the samples from the bulls in the OG. This was probably because the bulls in the OG had more crosslinking of connective tissues than the animals in the YG. This crosslinking of connective tissues contributes to the toughness of meat from older animals. Additionally, the average NCBS value for bulls in the YG was 3.8% lower than that for bulls in the OG. Likewise, Mojto et al. [26] and Nogalski et al. [22] also reported insignificant differences in the panel assessment of the sensory parameters of meat between younger and older bulls. The effects of muscle type on the sensory panel scores, such as juiciness and acceptability, were statistically significant (*p* < 0.01). Although the tenderness, palatability and flavor intensity scores that were determined for the LD muscle samples were greater than those determined for the GM samples, the differences were not statistically significant. Although the levels of free amino acids, nucleotides and minerals that contribute to the taste of meat were not measured in the present study, the aforementioned results could be attributed to the relatively higher amounts of these compounds in the LD muscle compared to the GM muscle [27]. The results of the present study were also in agreement with the findings of Kopuzlu et al. [3], who noted that the sensory characteristics of the LD muscle were higher than those of the GM muscle.

Textural profile analyses of meats focus on assessing differences in tenderness or toughness. Textural profile analyses are used to evaluate the texture of meat and have the advantage of being able to assess multiple variables, such as hardness, cohesiveness, springiness and chewiness [28]. Additionally, panel assessments also offer a superior indicator of meat tenderness compared to WBSF value [29]. In the present study, none of the differences in the parameters of the TPA between the slaughter age groups were statistically significant. The hardness of the meat from bulls in the OG was 6.49% lower than that of the meat from bulls in the YG, but this difference was not statistically significant. The higher hardness TPA value of the meat from bulls in the YG could have been due to insufficient muscle fattening as the samples became more tender with the increasing intramuscular fat content that was associated with advanced slaughter age [30,31,32]. However, the TPA results also revealed that muscle type had significant impacts on the hardness, gumminess and chewiness of the meat and that the values of the TPA parameters in the OG were lower than those in the YG. The findings of the current study agreed with those of Kopuzlu et al. [3], who reported that slaughter age had significant effects on the hardness, gumminess and chewiness of meat.

The effects of slaughter age (17 vs. 22 months) and muscle type (LD vs. GM) on the WBSF value did not differ significantly, i.e., our instrumental evaluation of tenderness using WBSF devices, as with the TPA results, demonstrated that the meat from bulls in both groups required similar maximum cutting forces. These findings were in agreement with the results of the studies carried out by Sargentini et al. [33], Bures and Barton [21] and Nogalski et al. [22]. However, the WBSF value of the LD muscle samples was lower than that of the GM muscle samples, although the difference was not statistically significant. Similarly, the LD and GM muscles were reported as "tender muscles" by Belew et al. [34], who also found that the average WBSF value of the LD muscle was slightly lower than that of the GM muscle.

In general, meat consumers are becoming more conscious and more aware of the impact of their diets on their health (especially the risk of atherosclerosis), which has increased consumer interest in the properties and nutritional value of meat (especially fatty acid composition). Furthermore, fatty acid profiles also determine meat quality because the fatty acid composition of muscle and adipose tissues determines the oxidative stability of the muscle and the firmness/oiliness of the adipose tissues, which in turn affects the flavor and color of the muscle [7].

The fatty acid composition of meat is important for human health reasons. Furthermore, fatty acid profiles also have crucial effects on meat quality, including the firmness of fat tissues, color, shelf life and flavor [7]. Our findings regarding fatty acid composition demonstrated that there were significant differences in palmitic acid (C16:0), linoleic acid (C18:2) and linolenic acid (C18:3) content between the slaughter age groups (Figure 1). Additionally, palmitic acid, stearic acid, oleic acid (C18:1n9c) and linoleic acid (C18:2) were the predominant fatty acids in the fat samples. These results concurred with the findings of Humada et al. [35] and Ugarkovic et al. [8].

The amount of SFA in beef has negative nutritional significance since palmitic acid and myristic acid (C14:0) raise serum cholesterol, whereas stearic acid (C18:0) has a neutral effect on LDL cholesterol levels [36]. The percentages of palmitic acid (C16:0) and stearic acid (C18:0) were the highest out of the SFAs in this study (Figure 1). Likewise, Raes et al. [37] and Momot et al. [38] also reported that palmitic acid and stearic acid were the major SFAs in the intramuscular fatty acid profiles in their works. In the present study, slaughter age did not cause any significant differences in individual or total SFAs, except for palmitic acid (C16:0), i.e., the total SFA content did not significantly alter with increasing slaughter age (Figure 2). These results were in agreement with the findings of Sargentini et al. [33] and Humada et al. [35].

Although the total MUFA percentage of the meat from the Holstein Friesian bulls slightly decreased with increasing slaughter age, the effects of slaughter age on the total MUFAs were not statistically significant. These outcomes were in line with those of the studies conducted by Ugarkovic et al. [8] and Nogalski et al. [22].

Polyunsaturated fatty acids are considered to improve lipid markers by decreasing plasma triacylglycerols and ApoB-100, which in turn decreases the level of LDL cholesterol [39]. In the present study, the concentrations of PUFAs, the PUFA/SFA ratio and the percentages of n-6 fatty acids significantly decreased with increasing slaughter age (Figure 2). This result could be attributed to the dynamics of changes in the muscle tissues as PUFAs are lower in the muscles of older animals [40]. In contrast to the current study, De Freitas et al. [41] and Pogorzelska-Przybyłek et al. [22] reported lower percentages of SFAs, similar percentages of MUFAs and considerably higher percentages of PUFAs in beef cattle steers and crossbred beef bulls.

Higher PUFA/SFA ratios are considered desirable for human nutrition [42]. The PUFA/SFA ratio in the YG was 10.8% higher than that in the OG, and the ratio decreased with increasing slaughter age. These results were in accordance with the findings of Ugarkovic et al. [8] and Pogorzelska-Przybyłek et al. [23]. The minimum recommended PUFA/SFA ratio for the human diet is 0.45. The PUFA/SFA ratio values determined in this study were considerably lower (0.064–0.074) due to the low PUFA concentrations in the analyzed meat samples.

The carcasses of the Holstein Friesian bulls in the YG contained fewer OFAs and more DFAs. Additionally, the DFA/OFA ratio increased significantly with decreasing slaughter age, i.e., the carcasses of younger Holstein Friesian bulls had more desirable DFA/OFA ratios (higher values) than the carcasses of the bulls in the OG.

In the present study, the percentages of SFAs in internal fat tissues were significantly (*p* < 0.01) higher than those in subcutaneous fat and intramuscular fat deposits. These results agreed with the findings of Sobczuk-Szul et al. [43], who noted that the percentages of SFAs in the visceral, intermuscular, intramuscular and subcutaneous fat tissues of bull carcasses were 59.6%, 57.6%, 49.6% and 46.8, respectively. Likewise, Aldai et al. [44] also reported that higher SFA concentrations were found in intermuscular fat tissues compared to intramuscular and subcutaneous fat tissues.

In the present study, the highest amounts of MUFAs were found in intramuscular and subcutaneous fat tissues as opposed to internal fat tissues. However, Sobczuk-Szul et al. [43] revealed that intramuscular fat had a higher MUFA concentration (46.2%) than visceral fat (36.7%) and intermuscular fat (38.7%) but a lower MUFA concentration than subcutaneous fat. On the other hand, Aldai et al. [44] noted that intramuscular fat tissues had a lower MUFA value (33.3%) compared to intermuscular fat deposits (39.9%) and subcutaneous fat deposits in the carcasses of yearling bulls.

The DFA and OFA content and the DFA/OFA ratio were all significantly affected by fat type. The highest percentage of OFAs was found in subcutaneous fat tissues rather than intramuscular fat or internal fat. The healthiest DFA/OFA ratios were obtained from intramuscular and internal fat deposits. However, the UFA/SFA ratio of internal fat was the lowest among those of all considered fat types. In addition, the most desirable UFA/SFA ratio was obtained from intramuscular fat. These results were consistent with the findings of Aldai et al. [44], who found the highest UFA/SFA ratios in the intramuscular fat tissues of yearling bulls from different genetic groups.

The PUFA/SFA ratio is an index that is normally used to assess the nutritional value of fat, and the target for the human diet is 0.45. Fats that have low PUFA/SFA ratios are considered unfavorable since they may lead to increases in blood cholesterol levels. Fats from ruminants normally have PUFA/SFA values that are below this recommendation [45]. The present study found that the highest PUFA/SFA ratio was in intramuscular fat tissues compared to subcutaneous and internal fat tissues. The PUFA/SFA ratio of intramuscular fat was 15.6% and 8.8% higher than those of subcutaneous fat and internal fat, respectively. Similarly, Sobczuk-Szul et al. [43] and Aldai et al. [44] noted that the PUFA/SFA ratio of intramuscular fat (LD) was superior to those of subcutaneous fat, intermuscular fat and visceral fat.

## 5. Conclusions

The current study demonstrated that the Holstein Friesian bulls in the YG were characterized by higher panel tenderness scores and OFA content. Furthermore, the panel tenderness score, PUFA content and PUFA/SFA and DFA/OFA ratios of the meat samples from the bulls decreased significantly with increasing slaughter age, whereas the OFA content increased significantly (*p* < 0.05) with increasing slaughter age. However, older animals had higher protein content compared to younger animals. The meat from the bulls in the OG was considered to be less healthy in terms of fatty acid profiles and nutritional indices, such as PUFA/SFA and the DFA/OFA ratios, compared to the meat from animals in the YG. Moreover, intramuscular fat (LD) and internal fat had high PUFA percentages, SFA content, PUFA/SFA and DFA/OFA ratios; however, the OFA percentages of internal and intramuscular fat deposits decreased with increasing slaughter age. In conclusion, this study clearly indicated that slaughter age, muscle type and fat type were significant sources of variations in the textural characteristics, chemical composition, sensory panel scores and fatty acid profiles of meat; therefore, these factors should be considered when evaluating meat from Holstein Friesian bulls that are intended for human consumption.

## Figures and Tables

**Table 1:** Chemical composition of the GM and LD muscles of bulls slaughtered at different ages expressed as least squares means ± standard errors.

	Slaughter Age (SA)		Muscle (M)		Significance	
	YG (*n* = 13)	OG (*n* = 13)	GM (*n* = 26)	LD (*n* = 26)	SA	M
Crude Protein	21.33 ± 0.13	21.84 ± 0.13	21.16 ± 0.13	22.01 ± 0.13	*p* < 0.01	*p* < 0.01
Ash Content	1.12 ± 0.01	1.12 ± 0.01	1.09 ± 0.01	1.14 ± 0.01	ns	*p* < 0.01
Crude Fat	1.46 ± 0.11	1.60 ± 0.11	1.46 ± 0.11	1.60 ± 0.11	ns	ns
Moisture	75.44 ± 0.18	75.29 ± 0.18	75.62 ± 0.18	75.10 ± 0.18	ns	*p* < 0.05

YG: young group, OG: old group, GM: *Gluteus medius*, LD: *Longissimus dorsi*, *p* < 0.05: significant, *p* < 0.01: highly significant, ns: non-significant.

**Table 2:** Sensory panel scores and number of chews before swallowing of LD and GM muscles of the Holstein Friesian bulls expressed as least squares means ± standard errors.

	Slaughter Age (SA)		Muscle (M)		Significance	
	YG (*n* = 13)	OG (*n* = 13)	GM (*n* = 26)	LD (*n* = 26)	SA	M
Tenderness	6.06 ± 0.199	5.56 ± 0.199	5.65 ± 0.199	5.97 ± 0.199	*p* < 0.05	ns
Juiciness	5.77 ± 0.148	5.73 ± 0.148	5.47 ± 0.148	6.03 ± 0.148	ns	*p* < 0.01
Acceptability	6.38 ± 0.38	6.11 ± 0.138	6.00 ± 0.138	6.48 ± 0.138	ns	*p* < 0.01
Palatability	6.06 ± 0.147	5.93 ± 0.147	5.83 ± 0.147	6.16 ± 0.147	ns	ns
Flavor Intensity	5.99 ± 0.146	5.88 ± 0.146	5.81 ± 0.146	6.06 ± 0.146	ns	ns
NCBS	30.94 ± 1.24	32.11 ± 1.24	32.64 ± 1.24	30.41 ± 1.24	ns	ns

YG: young group, OG: old group, GM: *Gluteus medius*, LD: *Longissimus dorsi*, *p* < 0.05: significant, *p* < 0.01: highly significant, NCBS: number of chews before swallowing ns: non-significant.

**Table 3:** The TPA scores and WBSF values (lb.) of the GM and LD muscles of bulls slaughtered at different ages, given as least squares means ± standard errors.

	Slaughter Age (SA)		Muscle (M)		Significance	
	YG (*n* = 13)	OG (*n* = 13)	GM (*n* = 26)	LD (*n* = 26)	SA	M
Hardness	85.23 ± 3.87	80.03 ± 3.87	99.26 ± 3.87	65.99 ± 3.87	ns	*p* < 0.01
Adhesiveness	0.130 ± 0.051	0.174 ± 0.051	0.108 ± 0.051	0.195 ± 0.051	ns	ns
Resilience	0.131 ± 0.004	0.129 ± 0.004	0.131 ± 0.004	0.129 ± 0.004	ns	ns
Cohesiveness	0.403 ± 0.007	0.410 ± 0.007	0.402 ± 0.007	0.411 ± 0.007	ns	ns
Springiness [18]	4.42 ± 0.12	4.50 ± 0.12	4.48 ± 0.12	4.44 ± 0.12	ns	ns
Gumminess [18]	34.31 ± 1.7	32.42 ± 1.7	39.67 ± 1.7	27.07 ± 1.7	ns	*p* < 0.01
Chewiness [19]	149.74 ± 9.03	148.82 ± 9.03	177.46 ± 9.03	121.11 ± 9.03	ns	*p* < 0.01
WBSF [20]	13.00 ± 0.49	12.91 ± 0.49	13.14 ± 0.49	12.77 ± 0.49	ns	ns

YG: young group, OG: old group, GM: *Gluteus medius*, LD: *Longissimus dorsi*, *p* < 0.05: significant, *p* < 0.01: highly significant, ns: non-significant.

## Data Availability

The data will be made available from the corresponding author upon request.
